# Formation rate of secondary anal fistula after incision and drainage of perianal Sepsis and analysis of risk factors

**DOI:** 10.1186/s12893-020-00762-3

**Published:** 2020-05-06

**Authors:** Zongqi He, Jun Du, Kaiwen Wu, Jiajia Chen, Bensheng Wu, Jianhua Yang, Zhizhong Xu, Zhihui Fu, Li Pan, Ke Wen, Xiaopeng Wang

**Affiliations:** 1 Department of Anorectal Surgery, Suzhou Hospital of Traditional Chinese Medicine Affiliated to Nanjing University of Chinese Medicine, No. 18, Yangsu Road, Suzhou, Jiangsu China; 2 Department of Anorectal Surgery, Kunshan Fourth Peoples Hospital, No. 21, Zhenbei Road, Kunshan, Jiangsu China; 3 Department of Radiology, Suzhou Hospital of Traditional Chinese Medicine Affiliated to Nanjing University of Chinese Medicine, No. 18, Yangsu Road, Suzhou, Jiangsu China

**Keywords:** Perianal sepsis, Anal fistula, Secondary, Incision and drainage

## Abstract

**Background:**

The choice of surgery for perianal sepsis is currently controversial. Some people advocate one-time radical surgery for perianal sepsis, while others advocate incision and drainage. The objective of this study is to observe the formation probability of secondary anal fistula after incision and drainage in patients with perianal sepsis and determine factors that contribute to secondary anal fistula after incision and drainage.

**Methods:**

A retrospective descriptive analysis was conducted in 288 patients with perianal sepsis who were treated with anorectal surgery in the Suzhou Hospital of Traditional Chinese Medicine from January 2016 to June 2018. The patients were followed by telephone, physical examination, and pelvic MRI examination for at least 1 year after surgery.

**Results:**

Three patients were not followed, 98 patients did not receive surgical treatment or one-time radical surgery for perianal sepsis, and 187 patients were ultimately identified for the study. Anal fistula was present in 105 patients, and the rate of formation of secondary anal fistula was 56.15%. There was no statistically significant difference in the fistula formation rate between different types of sepsis (*P*>0.05). And, in patients with secondary anal fistula, there was no significant correlation between the location of sepsis and the type of secondary anal fistula (*P*>0.05).

**Conclusions:**

The incidence of secondary anal fistula after incision and drainage of perianal sepsis is 56.15%, which is lower than the incidence found in previous study. Young is a risk factor for secondary anal fistula after incision and drainage of perianal sepsis. There is no significant correlation between the location of sepsis and the type of secondary anal fistula. Simple incision and drainage is a suitable choice for patients with acute perianal sepsis.

## Background

Perianal sepsis is a common acute disease in the field of colorectal surgery [1]. In China, the performance of one-time radical surgery for perianal sepsis in patients with this disease is general practice for most surgeons [2–7]. Abscessectomy is typically performed for lower sepsis, and cutting seton is typically placed for upper sepsis [7]. The advantage of this treatment is the reduction in the formation rate of postoperative secondary anal fistula [2–7]; however, at the same time, this treatment causes sphincter function injury and fecal incontinence in some patients [7].

Furthermore, a recent study in the United Kingdom with a large sample size found that the formation rate of secondary anal fistula after incision and drainage of common perianal sepsis was 15.5%, which is far lower than what had been previously thought [8]. However, this reliable formation rate lacks relevant research data in China.

The treatment of perianal sepsis in the anorectal surgery department of the Suzhou Hospital of Traditional Chinese Medicine is as follows: incision and drainage are performed during the acute stage, and fistulectomy or fistulotomy is performed if secondary anal fistula occurs; otherwise, patients continue to receive observation. This study retrospectively descriptively analyzed the incidence of secondary anal fistula after incision and drainage in 187 patients with perianal sepsis treated in the anorectal surgery department of the Suzhou Hospital of Traditional Chinese Medicine, and analyzed the possible risk factors of secondary anal fistula formation after incision and drainage of perianal sepsis.

## Methods

From January 2016 to June 2018, 288 patients with perianal sepsis diagnosed and treated in the anorectal surgery department of the Suzhou Hospital of Traditional Chinese Medicine were obtained by searching the case database. The diagnosis and surgical records were verified by an experienced anorectal surgeon in our hospital. If the patient also had inflammatory bowel disease (IBD), these data were recorded. If perianal sepsis in the patient was caused by foreign body stab wound infection, these data were excluded. At the same time, patients with pilonidal abscesses and diabetes were not included. Except for the cases that needed to be excluded as described above, of the remaining cases, the patients who underwent incision and drainage were included in this study.

During this study, all patients were treated by a standard protocol. All patients were drained by means of a single radial incision under local or spinal anesthesia shortly after arrival on the ward. If the abscess was too large, multiple radial incisions may be made and drained using loose seton. Preoperative imaging, Computed Tomography (CT) or Magnetic Resonance Imaging (MRI), was necessary before making an incision while the surgeon suspected that the abscess was above the levator ani muscle. An antibiotic was given intravenously continuously for 6 days after surgery. Chinese medicine baths (Waike Zuoyu formula) were begun on the first postoperative day and were continued until the wounds were healed.

Follow-up was initiated in July 2019. The follow-up was conducted by an anorectal surgeon who was not involved in the operation from another hospital and a nurse specialist who was experienced with follow-up in our hospital. The postoperative follow-up time of all the included patients was more than one year. Patients were followed by telephone to see if there was any perianal pain or purulent discharge. If none of the symptoms described above occurred or if pelvic MRI indicated no abnormalities, patients were defined as having no anal fistula formation. If the patient had perianal swelling and pain or discharge pus symptoms, and in our hospital or other hospitals, the physical examination and/or pelvic MRI suggested anal fistula, the patient was defined as having anal fistula formation [9]. If the patient had received pelvic CT or MRI examination in our hospital, 2 experienced radiologists and 1 anorectal surgeon read the film independently to identify the fistula location. When there were differences in opinions, 3 people would discuss together and provide final opinions of the images.

### Statistical analysis

All statistical analyses were carried out using SPSS® version 21.0 (SPSS, Inc., New York, IBM). Continuous variables were analyzed by *t-*tests or nonparametric tests, and classified variables were analyzed by the chi-square test. The correlation was analyzed by binary logistic regression. *P* < 0.05 was statistically significant.

## Results

**Fig. 1:**
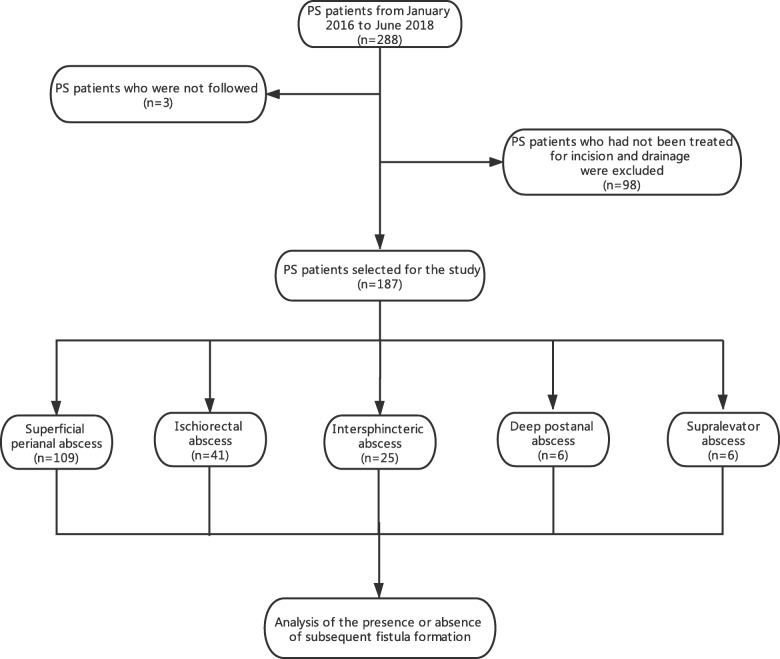
Flow chart of the data analysis for patients with perianal sepsis

Among the 288 patients with perianal sepsis, 3 patients were not followed (incorrect contact information), and 98 patients did not receive surgery or one-time radical surgery for perianal sepsis. Ultimately, a total of 187 patients were effectively followed and included in this study for statistical analysis. The research flow chart is shown in Fig. 1.

### General data analysis of patients with perianal sepsis

Among the 187 patients with perianal sepsis, 158 were male and 29 were female, and the incidence ratio of males to females was 5.45:1. The age at perianal sepsis onset ranged from 10 years to 70 years, with a median age of 35 years. The longest duration was 30 days, the shortest duration was 2 days, and the median duration was 5 days. Among the cases of sepsis, there were 109 cases of superficial perianal sepsis, 6 cases of deep postanal sepsis, 41 cases of ischiorectal sepsis, 25 cases of intersphincteric sepsis and 6 cases of supralevator sepsis. IBD was also present in 8 patients. The follow-up time ranged from 15 months to 44 months, with a median follow-up time of 28 months. General information is shown in Table 1.

**Table 1:** Clinical characteristics of the study patients. Values are expressed as the mean ± standard deviation, number, or median (Q1-Q3)

	Total	With Fistula	Without Fistula	*P*-value
Patients(n)	187	105	82	–
Gender(n[%])	187 [100]	105 [100]	82 [100]	0.908
Age (years)	35 (29–47)	34 (29–45)	37 (31–49)	0.038
Abscess duration (days)	5 (4–7)	5 (3.5–7.0)	5 (3.8–7.0)	0.638
Abscess location(n[%])				
Superficial perianal abscess	109 [58.3]	58 [55.2]	51 [62.2]	0.480
Deep postanal abscess	6 [3.2]	2 [1.9]	4 [4.9]	
Ischiorectal abscess	41 [21.9]	27 [25.7]	14 [17.1]	
Intersphincteric abscess	25 [13.4]	14 [13.3]	11 [13.4]	
Supralevator abscess	6 [3.2]	4 [3.8]	2 [2.4]	
With IBD(n[%])	8 [4.3]	6 [5.7]	2 [2.4]	0.272
Time of follow-up (months)	28 (22–35)	27 (21.0–34.5)	28 (23–36)	0.408

### Fistula formation after incision and drainage of perianal sepsis

As shown in Tables 1, 105 of 187 patients (56.15%) with perianal sepsis who underwent incision and drainage experienced the formation of anal fistula. According to the analysis of the different locations of sepsis, the incidence of fistula in the group with superficial perianal sepsis was 53.21%, and the incidence of fistula in the group with deep postanal sepsis, ischiorectal sepsis, intersphincteric sepsis, and supralevator sepsis was 33.33, 65.85, 56, and 66.67%, respectively. There was no significant difference in secondary fistula formation among different sepsis locations (*P* = 0.48, > 0.05).

### Univariate analysis of anal fistula formation

**Fig. 2:**
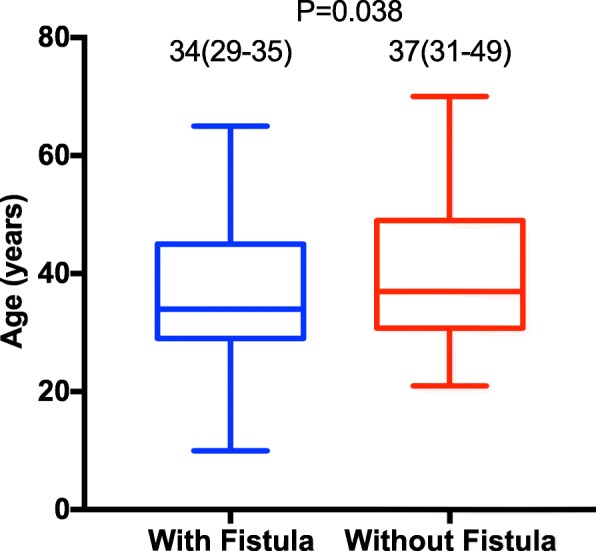
Relationship between secondary anal fistula after perianal sepsis incision and drainage and age

As shown in Table 1 and Fig. 2, after the analysis of the data of patients with perianal sepsis, in addition to the age at sepsis onset, which was correlated with postoperative anal fistula formation (*P* = 0.038, *P* < 0.05), there was no significant correlation between factors including patient sex (*P* = 0.908, > 0.05), sepsis duration (*P* = 0.638, > 0.05), sepsis location (*P* = 0.480, > 0.05), and time of follow-up (*P* = 0.408, > 0.05).

### Multivariate analysis of anal fistula formation

As shown in Table 2, multiple logistic regression analysis was performed to examine whether the location of perianal sepsis was an independent risk factor for anal fistula formation after incision and drainage of perianal sepsis. In the model, sex, age at onset, duration of disease, sepsis location, combined with IBD and follow-up time were included. The results showed that the location of sepsis was not an independent risk factor for secondary anal fistula after incision and drainage of perianal sepsis (odds ratio: 0.929, 95% confidence interval [CI]: 0.778–1.108, *P* = 0.411). At the same time, sex (odds ratio: 1.147, 95% confidence interval [CI]: 0.505–2.610, *P* = 0.743), duration (odds ratio: 0.971, 95% confidence interval [CI]: 0.913–1.033, *P* = 0.348), combined with IBD (odds ratio: 1.838, 95% confidence interval [CI]: 0.503–2.603, *P* = 0.748) 1.837, 95% confidence interval [CI]: 0.344–9.826, *P* = 0.476) and follow-up time (odds ratio: 1.009, 95% confidence interval [CI]: 0.972–1.048, *P* = 0.632) were not significantly associated with the presence of anal fistula after incision and drainage of perianal sepsis. However, age (odds ratio: 1.028, 95% confidence interval [CI]: 1.002–1.054, *P* = 0.035) was significantly associated with anal fistula formation after incision and drainage of perianal sepsis.

**Table 2:** Multiple logistic regression analysis of factors associated with secondary anal fistula after perianal sepsis incision and drainage

	Model		
	OR	95% CI	***P***-value
Sex(n[%])	1.147	0.505–2.610	0.743
Age (years)	1.028	1.002–1.054	0.035
Abscess duration (days)	0.971	0.913–1.033	0.348
Abscess location(n[%])	0.929	0.778–1.108	0.411
With IBD(n[%])	1.838	0.344–9.826	0.476
Time of follow-up (months)	1.009	0.972–1.048	0.632

### Relationship between sepsis location and type of anal fistula

**Fig. 3:**
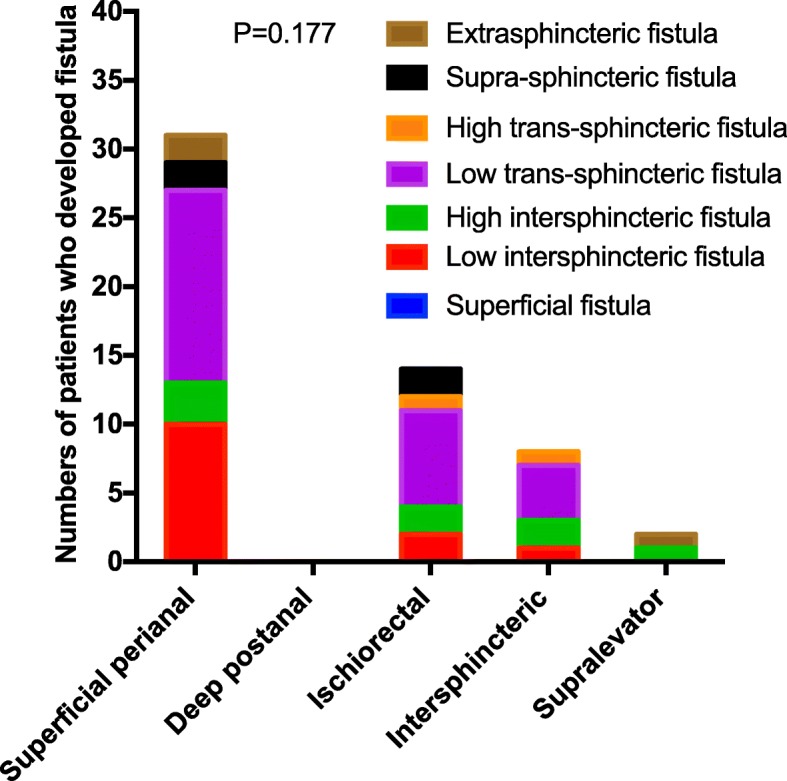
Relationship between perianal sepsis and secondary anal fistula

According to further analysis of the data from 105 patients with perianal sepsis who experienced the formation of anal fistula, 55 of them were re-examined or underwent surgery in our hospital, and the type of secondary anal fistula could be determined, while that of the other 50 patients could not be determined. Among the 55 patients, 31 had superficial perianal sepsis: 10 had low intersphincteric fistula, 14 had low trans-sphincteric fistula, 3 had high intersphincteric fistula, 2 had supra-sphincteric fistula, and 2 had extrasphincter fistula. In 14 patients with ischiorectal sepsis, half of them experienced formation of lower trans-sphincter fistula. There were 8 patients with intersphincteric sepsis, 50% of whom experienced the formation of lower trans-sphincteric fistula. There were 2 patients with supralevator sepsis, 1 of whom experienced secondary formation of high intersphincteric fistula, and the other experienced the formation of extrasphincter fistula. After analysis, there was no significant correlation between the location of sepsis and the type of secondary anal fistula (*P* = 0.177, > 0.05), as shown in Table 3 and Fig. 3*.*

**Table 3:** Relationship between perianal sepsis and secondary anal fistula

Abscess location	Total	Superficial fistula	Low intersphincteric fistula	High intersphincteric fistula	Low trans-sphincteric fistula	High trans-sphincteric fistula	Supra-sphincteric fistula	Extrasphincteric fistula	***P***-value
**Superficial perianal**	31	0	10	3	14	0	2	2	0.177
**Deep postanal**	0	0	0	0	0	0	0	0	
**Ischiorectal**	14	0	2	2	7	1	2	0	
**Intersphincteric**	8	0	1	2	4	1	0	0	
**Supralevator**	2	0	0	1	0	0	0	1	
**Total**	55	0	13	8	25	2	4	3

## Discussion

Perianal sepsis is one of the common diseases in the field of colorectal surgery, and it often causes substantial pain in individuals with the disease [1, 10, 11]. Perianal sepsis often occurs acutely. Once sepsis is formed, it is often difficult to heal on its own, and the condition changes rapidly. If perianal sepsis is not treated, it may cause inflammation and may spread [1, 10, 11]. The disease generally requires emergency surgery. The Italian Society of Colorectal Surgery (ISCR) recommends that perianal sepsis should be drained promptly [10].

However, in China, there are two surgical treatments for perianal sepsis: incision drainage and one-time radical surgery, but there is controversy. Some scholars believe that the traditional incision and drainage method is simple, can promptly improve the clinical symptoms of patients and does not damage sphincter function. However, other scholars believe that the surgical treatment of perianal sepsis should primarily be one-time radical surgery. The latter group believes that perianal sepsis is caused by glandular infection. In these low quality studies, approximately 18.18% ~ 66.67% of patients with perianal sepsis will form anal fistula after incision and drainage in China [2–7]. Mainly, for the treatment of sepsis involving the extent of the lesion, there are potential risks; if the patient also has tuberculosis, Crohn’s disease or other diseases, the probability of secondary anal fistula formation is greatly increased [1, 8]. Direct resection of low sepsis and the use of a cutting seton for upper sepsis can avoid recrudescence and anal fistula formation [7].

However, the ensuing problem is that some patients suffer from damage to the external sphincter, thus resulting in varying degrees of fecal incontinence [4, 7, 12]. It is possible that the proportion of patients with anal fistula who undergo radical resection is decreasing; however, do some of these patients actually form anal fistula? At present, there is increasing evidence that the proportion of patients who experience the formation of secondary anal fistula after perianal sepsis is not very high [13–19]. A large sample of data from the United Kingdom found that the incidence of secondary anal fistula after perianal sepsis incision and drainage was 15.5% [8]. At present, there are no reliable data that support the study of secondary anal fistula in patients with sepsis surgery in China. Our team reviewed and analyzed 288 patients with perianal sepsis. The total fistula formation rate was 56.15%, and there was no statistically significant difference in the fistula formation rate between different types of sepsis. The study by Ghahramani et al. also found location of abscess did not show any association with development of fistula [18]. At the same time, in patients with secondary anal fistula, there was no significant correlation between the location of sepsis and the type of secondary anal fistula.

At present, with the continuous development of imaging technology, research has confirmed that MRI can accurately display the specific location and number of fistula, which provides a favorable reference value for surgery [10, 11, 20, 21]. MRI is now the gold standard for the diagnosis of anal fistula and perianal sepsis, but a large number of patients with perianal sepsis often have not been subjected to this test at an earlier time [12]. For high and complex cases of perianal sepsis, if surgeons perform radical surgery without the help of MRI, recurrence is difficult to avoid [22]. Our experience is as follows: If high or complex perianal sepsis is suspected, MRI should be performed before surgery and should be used to determine the spacing and branching involved in the infection.

In terms of the risk factors for secondary anal fistula, sex, disease duration, sepsis location, and follow-up time were not independent risk factors for secondary anal fistula, and age was an independent risk factor. A similar study also reached the same conclusion, and it found that age younger than 40 years was a high risk factor for chronic anal fistula or perianal sepsis recurrence after incision and drainage [23]. In this study, whether IBD was present was not an independent risk factor for secondary anal fistula, and in another large retrospective analysis, IBD was clearly identified as an independent risk factor for secondary anal fistula formation; thus, the presence of IBD should be given sufficient attention [1, 8]. In medical practice, we have observed that an increasing number of patients with perianal sepsis combined with IBD are prone to secondary anal fistula. In this study, it may be that the sample size was too small to reflect this difference.

In summary, the proportion of patients with secondary anal fistula after perianal sepsis incision and drainage is not as high as expected, and nearly half of patients will not develop anal fistula. If only used to prevent anal fistula formation or sepsis recurrence, arbitrarily performing radical surgery for perianal sepsis is not only considered excessive surgical treatment but also increases the risk of fecal incontinence and significantly reduces the patient’s postoperative quality of life [1, 24–26]. Although there is evidence that managing an associated fistula during the acute phase of perianal sepsis can reduce fistula recurrence, there is no enough consensus to support surgeons operating immediate fistula surgery at incision and drainage of perianal sepsis [1].

Therefore, for patients with acute perianal sepsis, it may be necessary to perform incision and drainage to improve the patient’s clinical symptoms firstly. Next, if there is secondary anal fistula formation, it is a wise choice to perform fistulectomy or fistulotomy after MRI examination.

This study is limited by its retrospective design and relatively small sample size. If there are more samples, the evidence that secondary anal fistula formation rate after incision and drainage of perianal sepsis may be more adequate.

## Conclusions

Regardless of the cause of the perianal abscess, immediate incision and drainage is standard treatment. About 50% of patients treated with incision and drainage for perianal sepsis will go on to develop anal fistulas. Young is a risk factor for secondary anal fistula after incision and drainage of perianal sepsis. There was no statistically significant difference in the fistula formation rate between different types of sepsis. For patients with acute perianal sepsis, it may be a suitable choice to perform incision and drainage to improve the patient’s clinical symptoms firstly.

## Data Availability

The data and materials during the current study are available from the first author and the corresponding author on reasonable request.

## Abbreviation

IBD: Inflammatory bowel disease
PS: Perianal sepsis
